# A Rare Transverse Colon Hiatal Herniation as a Complication of an Esophagectomy

**DOI:** 10.7759/cureus.72046

**Published:** 2024-10-21

**Authors:** Abby L Cummings, Nathaniel G Blanchard, Jenna Farnum, Tristan D Packard, Nicole L Geske, Libby J Bradley, William McMillan

**Affiliations:** 1 Radiology, Division of Human Anatomy, Michigan State University, East Lansing, USA; 2 Radiology, Michigan State University College of Osteopathic Medicine, East Lansing, USA

**Keywords:** anatomy, esophageal hiatal hernia, hiatal hernia, post-esophagectomy hernia, prosection

## Abstract

Hiatal herniations involving the transverse colon are a rare condition. This case study explores the hiatal herniation of the transverse colon as a complication of an esophagectomy through the prosection findings of a 91-year-old male anatomical donor ("donor") who had a documented esophagectomy procedure due to esophageal adenocarcinoma. A thorough dissection of the abdomen and thorax confirmed that a large portion of the esophagus was removed during an esophagectomy, and the remaining cervical portion was reconnected to the stomach in the posterior mediastinum of the thoracic cavity. A type IV hiatal hernia involving the transverse colon and greater omentum was also noted. This study aims to evaluate the donor's noted esophageal hiatal herniations and how surrounding atypical anatomy may be correlated with the history of a completed esophagectomy.

## Introduction

In normal anatomy, the esophagus passes through the esophageal hiatus along with the anterior and posterior vagal trunks, lymphatic vessels, and esophageal branches of the left gastric artery and vein [1]. The esophageal hiatus forms a functional constriction of the esophagus [1]; however, the stomach or other abdominal organs may herniate through the hiatus. These hiatal hernias are classified into four types. The most frequent is type I (sliding), where the lower esophageal sphincter (LES) and a small proximal portion of the stomach enter into the thoracic cavity [2]. Type II (rolling or paraesophageal) is when the stomach (fundus region) enters the thoracic cavity adjacent to the esophagus. In this type, the LES remains in its normal position within the abdominal cavity [2]. Type III is where the LES and stomach fundus are intrathoracic [2]. Type IV is characterized by the herniation of the stomach and another organ into the thorax [3]. 

Esophagectomy

When the esophagus takes on a disease such as cancer, whether benign or malignant, the treatment most commonly used is an esophagectomy, which is the partial or full removal of the esophagus [4]. From a surgical perspective, an esophagectomy is a complex case with various techniques used depending on the patient's needs. Common techniques include a transhiatal, Ivor Lewis, McKeown, and thoracoabdominal esophagectomy. The transhiatal esophagectomy utilizes an incision in the neck (anterior to the sternocleidomastoid muscle) and abdomen to approach the esophageal region [5]. In the Ivor Lewis approach, an incision is made on the right side of the chest and abdomen. The McKeown approach combines these techniques by using an incision through the neck, chest, and abdomen. Finally, the thoracoabdominal esophagectomy requires two incisions: one substantial incision on the left side spanning from the chest to the abdomen and another smaller incision in the neck [6]. 

As stated earlier, an esophagectomy, regardless of surgical approach, is a complex case and poses risks to the patient. According to Edmondson et al., surgical complications vary from early to late-onset, while others can happen at any point following the surgery [3]. Early complications include recurrent laryngeal nerve palsy, chylothorax, pneumonia, aspiration, atrial dysrhythmias, and anastomotic leaks. Over a more extended period following surgery, patients can experience stricture, bile reflux, dumping syndrome, and malabsorption. Dysphagia, delayed gastric emptying, and reflux are complications noted as non-time sensitive and can happen at any time post-surgery [5]. Overall, atrial fibrillation and pneumonia are the most common side effects experienced following an esophagectomy [3]. 

To improve patient care and quality of life, it's paramount that patients and surgeons understand the potential surgical complications associated with esophagectomies so patients can make informed decisions about their care and surgeons can better refine their surgical approaches and technical skills. This study aimed to investigate possible surgical complications and outcomes observed following an esophagectomy and how resulting atypical anatomy can potentially complicate patient outcomes.

## Case presentation

**Figure 1:**
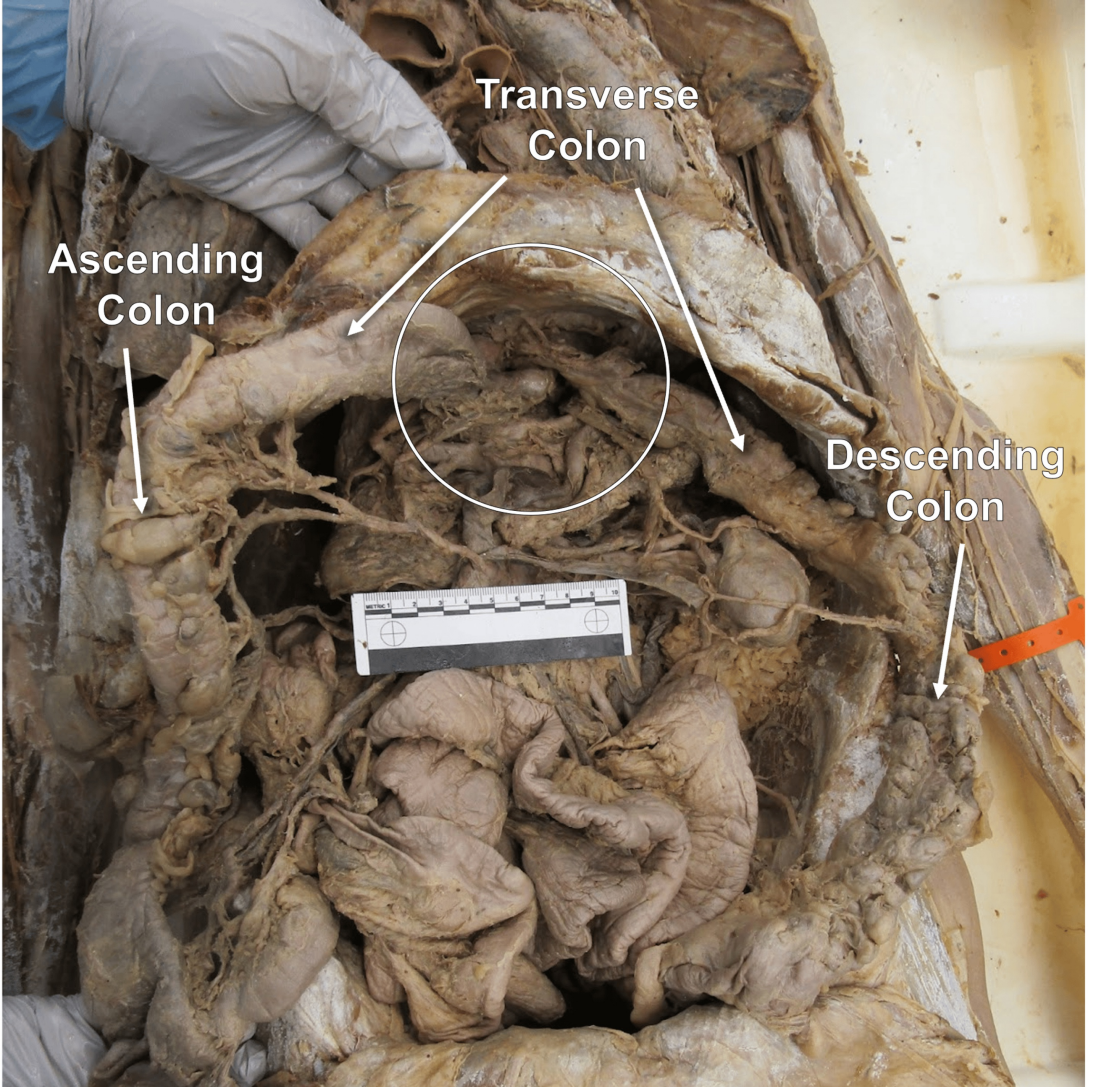
Anterior view of prosection demonstrating hiatal hernia Anterior view of abdominal prosection. The hiatal hernia involving the transverse colon is encircled. The greater omentum in the abdomen was removed. The ruler in the image measures 10 cm.

During a routine dissection of a 91-year-old male anatomical donor ("donor") through the Willed Body Program at Michigan State University, it was noted that the entire stomach, greater omentum, and part of the transverse colon and its mesentery (transverse mesocolon) were located in the posterior mediastinum and contained within a sac, hypothesized to be composed of mediastinal pleura. The sac measured 8.5 cm in width (lateral-medial) by 8 cm (superior-inferior). In the abdominal cavity, the mid-portion of the transverse colon and the transverse mesocolon were found herniated through the esophageal hiatus. Adhesions were found linking the transverse mesocolon to the diaphragm and part of the formed sac within the mediastinum. The herniated portion of the transverse colon could not be precisely measured due to the many adhesions in the mediastinal pleural sac; however, at least 11 cm of the transverse colon was found within the sac (Figure 1).

**Figure 2:**
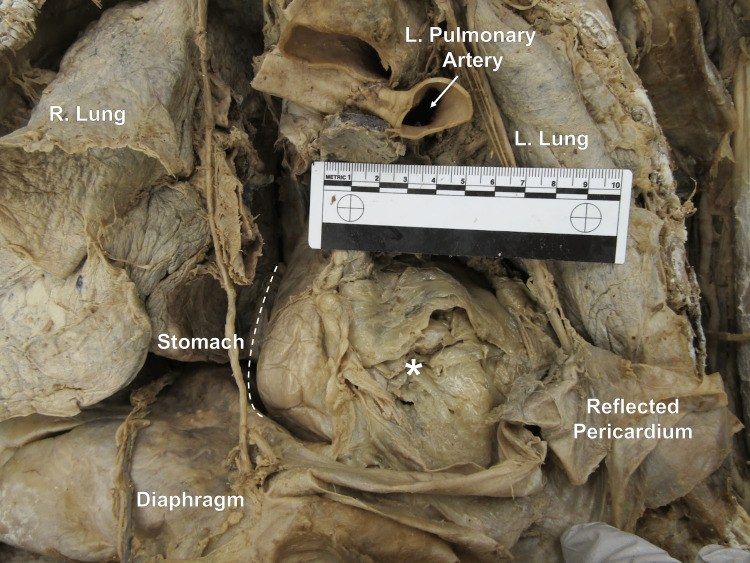
Anterior view of the thorax Anterior view of the thorax with the opened mediastinal pleural sac containing the greater omentum, the stomach, and a portion of the transverse colon. The ruler in the image measures 10 cm. * indicates the greater omentum.

A thorough review of the donor's past medical history revealed an esophagectomy due to esophageal adenocarcinoma. As a part of this procedure, likely a complete esophagectomy, it's believed that the stomach and greater omentum were surgically placed in the thoracic cavity. The stomach tissue extended from the mediastinal pleura to the pharynx. Further prosection revealed many adhesions between the stomach tissue (acting as the esophagus) and the anterior spinal column. Most of the transverse colon and its associated mesentery (the transverse mesocolon) were found located in the thoracic cavity and were contained within a mediastinal pleura-defined sac extending from the esophageal hiatus (Figure 2). 

Therefore, it is believed that the transverse colon subsequently herniated through the esophageal hiatus. Additionally, the donor's esophageal hiatus was enlarged with a diameter of 4.5 cm and 5 cm at its shortest and longest cross-sections, respectively. The average diameter of a normal esophageal hiatus without a hernia is 1.8 cm (±0.4 cm) at the longest cross-section and 1 cm (±0.3 cm) at its shortest cross-section [2].

**Figure 3:**
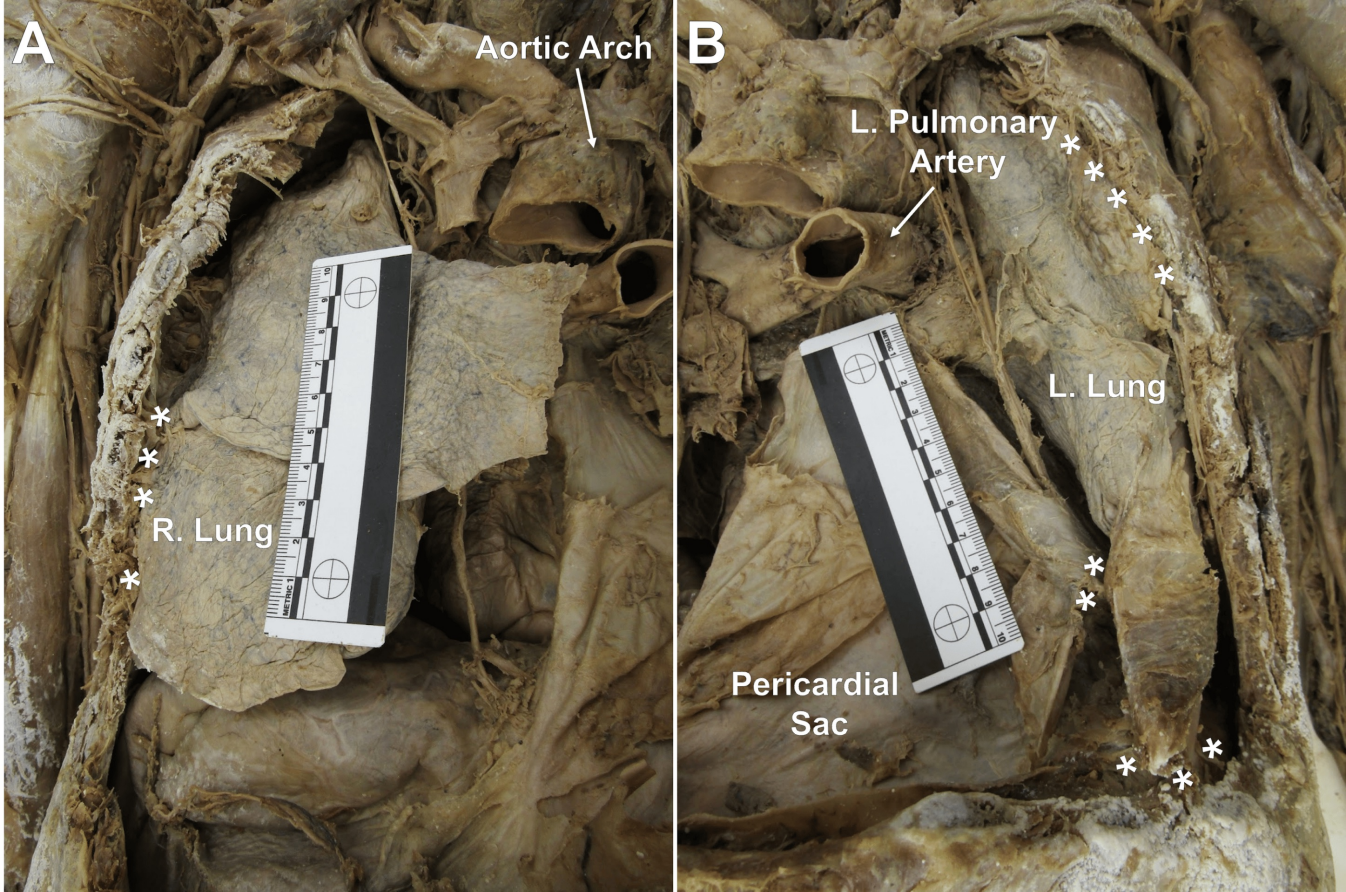
Lung abnormalities (A) Anterior view of the right lung. Adhesions were noted along the lower right lobe, attaching it to the inner thoracic wall. (B) Anterior view of the left lung. Numerous adhesions were noted between the upper left lobe to the inner thoracic wall and the lower left lobe to the pericardium and diaphragm. The ruler in both images measures 10 cm. * indicates observed adhesions.

Blunt dissection of the mediastinum in the thoracic cavity revealed adhesions of the fibrous pericardium to the left lung and bilateral adhesions on the lateral aspects of the lungs to the costal parietal pleura. Adhesions on the left lung were more numerous than on the right and extended to the base of the lung, which was adhered to the diaphragm (Figure 3).

**Figure 4:**
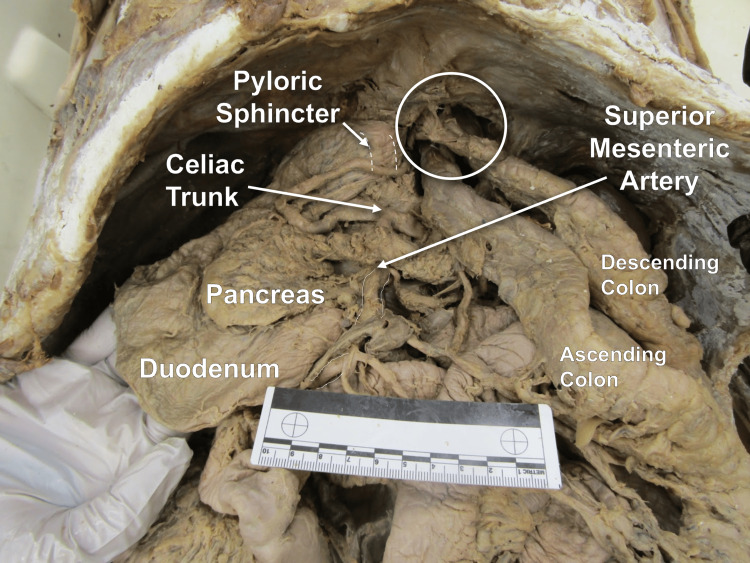
Anterior view of the superior abdomen Hiatal hernia involving the transverse colon encircled. Widening of the duodenum can also be seen. The ruler in the image measures 10 cm.

Additional gross anatomical findings included a significantly enlarged duodenum and variations in the abdominal vasculature. The widest portion of the duodenum measured 10 cm. The average diameter of a normal duodenum is approximately 2.48 cm [7]. The celiac trunk did not display a left gastric artery, and the superior mesenteric artery (SMA) was found extending from the celiac trunk (Figure 4). There was no evidence of surgical anastomosis between the celiac trunk and SMA, and therefore, it is likely this was a pre-existing anatomical variation. Although the left gastric artery was likely altered due to the surgery, the location of the SMA is likely congenital. Furthermore, a stricture in the bowel was noted where the SMA overlies the third part of the duodenum. 

## Discussion

**Figure 5:**
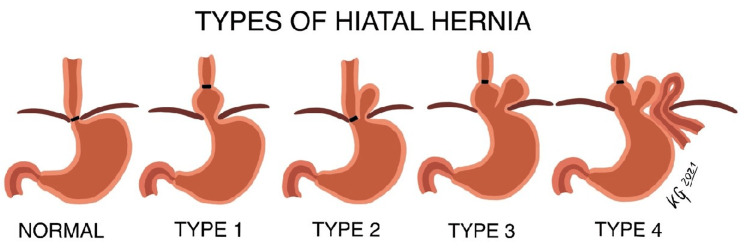
Types of hiatal hernia Diagrammatic representation of the normal versus esophageal hiatal hernia anatomy [8]. A type IV hernia was discovered in this prosected donor.

The anatomical abnormalities contained within this donor are proposed to illustrate the potential complications of an esophagectomy, supporting those commonly seen and establishing a pathogenesis hypothesis for a rare herniation. The limited medical history provided that this donor experienced common complications associated with an esophagectomy, including aspiration pneumonia and dysphagia. However, a rare complication observed in this donor was a herniation of the transverse colon through the esophageal hiatus. The authors’ approach to understanding this hernia was through the lens of a variation to a Type IV hiatal hernia. This type of hernia differs from other esophageal hiatal hernias as it includes the relocation of other abdominal organs besides the stomach (Figure 5).

The etiology of a type IV hiatal hernia is a lax esophageal hiatus, which can be caused by advanced age, smoking, obesity, or genetic disposition [9]. Another etiology is prolonged periods of increased intra-abdominal pressure, which can be caused by ascites, chronic cough, or chronic constipation [9]. Due to the abnormally enlarged esophageal hiatus (measured to be 4.5-5 cm in diameter), the authors hypothesize that this laxity was provoked by the donor's surgical history of an esophagectomy, allowing for the transverse colon and its associated mesentery to herniate through. The authors believe this herniation to be a complication, rather than an intentional maneuver, due to the presence of an overlying layer of what is presumed to be mediastinal pleura defining a sac in which the transverse colon was located within. This herniation appeared to incite mass effect-like complications resulting in a lateral compression of the left lung (observed in Figure 2 and Figure 3). 

Additional complications observed during our dissection noted a vast number of adhesions covering a majority of the viscera and parietal pleura within the thoracic cavity (Figure 3). These adhesions are likely due to an anastomotic leak involving the reconstructed esophagus, leading to intense inflammation and the end-stage tissue remodeling response, resulting in these findings [4]. Additionally, due to the invasive nature of an esophagectomy, adhesions are a probable byproduct of the significant manipulation of the involved regions [4]. 

The observed enlarged duodenum (at its widest point measured to be 10 cm) is hypothesized to be secondary to the known potential complication of dumping syndrome [4]. Dumping syndrome is a common complication of a variety of gastric-related surgeries and is characterized by alterations in the mechanisms of gastric emptying that lead to higher volumes of contents leaving the stomach and entering the small intestine [10]. Although the donor's limited medical history did not address the presence of dumping syndrome, the authors relied on anatomical cues to support their hypothesis, in addition to the association of dumping syndrome with gastric-related surgeries and the manifestation of persistent increased volume within the duodenum due to rapid gastric emptying and dysfunctional receptive reflexes [11,12]. Other possible causes for a dilated duodenum are adhesions, strictures, or obstructions possibly resulting from the herniation of the transverse colon that could lead to proximal dilations of the duodenum. Additionally, the path of the SMA appeared to be compressing the third portion of the duodenum and overlies a duodenal stricture. Similar findings are seen in SMA syndrome, where compression of the duodenum between the SMA and abdominal aorta may lead to proximal dilation of the duodenum due to luminal constriction caused by the compression [13]. SMA syndrome onset can have a variety of different causes, ranging from anatomical variants to previous gastric surgical procedures [13]. The anatomical variation of the SMA branching from the celiac trunk rather than directly from the abdominal aorta is believed to be congenital. This variation may have predisposed this donor to duodenal dilation through a mechanism similar to SMA syndrome. It may have been further exacerbated by the donor's surgical history [13]. 

A minimally invasive esophagectomy is a standard in treatment for esophageal adenocarcinoma, a condition consistent with the previous medical history of this donor. This procedure exposes patients to risks for a variety of early and late complications, including but not limited to aspiration pneumonia, atrial dysrhythmias, anastomotic leaks, dumping syndrome, dysphagia, delayed gastric emptying, and reflux [4]. The authors believe this donor suffered from a variety of these complications, indicated by the anatomical findings of vast collections of adhesions and an enlarged duodenum. The limited provided medical history of the donor listed two common complications associated with an esophagectomy: aspiration pneumonia and dysphagia. An additional rare complication of herniation of the transverse colon through the esophageal hiatus was found during dissection.

While hiatal herniation of the transverse colon following an esophagectomy has been found in the literature, the number of cases reported is deficient. The incidence of hiatal herniations occurring after an esophagectomy is approximately 2.8% [14-16]. However, many hiatal herniations are asymptomatic, so it can be hypothesized that this percentage does not include all cases. From the reported cases, the patient can undergo various symptoms, including dyspnea, nausea, abdominal pain, and general chest pain as they recover from the esophagectomy [14,15,17]. Diagnosis of the herniation can range from days to over two years post-esophagectomy [16]. 

## Conclusions

During a routine dissection of a 91-year-old male anatomical donor, it was discovered that the entire stomach was surgically placed in the thorax in an esophagectomy. The greater omentum and part of the transverse colon subsequently herniated through the esophageal hiatus into the thoracic cavity. Upon further examination, it was apparent that the herniation was only limited to the transverse colon with its associated mesentery and that the presence of the stomach within the thoracic cavity was a byproduct of a surgical history of an esophagectomy secondary to esophageal adenocarcinoma. 

An esophagectomy poses a risk for the manifestation of a variety of anatomical and physiological complications. This case study aims to contribute to the current literature on esophagectomy complications by providing a model illustrating some of the commonly observed complications as well as providing a hypothesis for a rare herniation of the transverse colon through the esophageal hiatus. This model should provide additional insight into the risks this procedure poses to educate patients, encourage modifications to the methodology of the operation, and improve the management of postoperative patients to minimize complications and improve patient outcomes. 
